# Multiorgan Development of Oxidative and Nitrosative Stress in LPS-Induced Endotoxemia in C57Bl/6 Mice: DHE-Based *In Vivo* Approach

**DOI:** 10.1155/2019/7838406

**Published:** 2019-05-22

**Authors:** Bartosz Proniewski, Agnieszka Kij, Barbara Sitek, Eric E. Kelley, Stefan Chlopicki

**Affiliations:** ^1^Jagiellonian University, Jagiellonian Centre for Experimetal Therapeutics (JCET), Bobrzynskiego 14, 30-348 Krakow, Poland; ^2^Jagiellonian University Medical College, Chair and Department of Toxicology, Medyczna 9, 30-688 Krakow, Poland; ^3^Department of Physiology & Pharmacology, West Virginia University, Morgantown, WV 26506, USA; ^4^Jagiellonian University Medical College, Chair of Pharmacology, Grzegorzecka 16, 31-531 Krakow, Poland

## Abstract

Detection of free radicals in tissues is challenging. Most approaches rely on incubating excised sections or homogenates with reagents, typically at supraphysiologic oxygen tensions, to finally detect surrogate, nonspecific end products. In the present work, we explored the potential of using intravenously (i.v.) injected dihydroethidine (DHE) to detect superoxide radical (O_2_^∙-^) abundance *in vivo* by quantification of the superoxide-specific DHE oxidation product, 2-hydroxyethidium (2-OH-E^+^), as well as ethidium (E^+^) and DHE in multiple tissues in a murine model of endotoxemia induced by lipopolysaccharide (LPS). LPS was injected intraperitoneally (i.p.), while DHE was delivered via the tail vein one hour before sacrifice. Tissues (kidney, lung, liver, and brain) were harvested and subjected to HPLC/fluorescent analysis of DHE and its monomeric oxidation products. In parallel, electron spin resonance (EPR) spin trapping was used to measure nitric oxide (^∙^NO) production in the aorta, lung, and liver isolated from the same mice. Endotoxemic inflammation was validated by analysis of plasma biomarkers. The concentration of 2-OH-E^+^ varied in the liver, lung, and kidney; however, the ratios of 2-OH-E^+^/E^+^ and 2-OH-E^+^/DHE were increased in the liver and kidney but not in the lung or the brain. An LPS-induced robust level of ^∙^NO burst was observed in the liver, whereas the lung demonstrated a moderate yet progressive increase in the rate of ^∙^NO production. Interestingly, endothelial dysfunction was observed in the aorta, as evidenced by decreased ^∙^NO production 6 hours post-LPS injection that coincided with the inflammatory burden of endotoxemia (e.g. elevated serum amyloid A and prostaglandin E_2_). Combined, these data demonstrate that systemic delivery of DHE affords the capacity to specifically detect O_2_^∙-^ production *in vivo*. Furthermore, the ratio of 2-OH-E^+^/E^+^ oxidation products in tissues provides a tool for comparative insight into the oxidative environments in various organs. Based on our findings, we demonstrate that the endotoxemic liver is susceptible to both O_2_^∙-^-mediated and nonspecific oxidant stress as well as nitrosative stress. Oxidant stress in the lung was detected to a lesser extent, thus underscoring a differential response of liver and lung to endotoxemic injury induced by intraperitoneal LPS injection.

## 1. Introduction

Reactive oxygen species (ROS) are critical components of various disease processes with superoxide anion radical (O_2_^∙-^) often assuming the center of attention. Due to the high reactivity of both ROS and reactive nitrogen species (RNS), most methods rely on the *ex vivo* quantification of stable end products or steady-state intermediates in tissues or biological fluids. Dihydroethidine (DHE) oxidation to superoxide-specific 2-hydroxyethidium (2-OH-E^+^), detected with high-performance liquid chromatography (HPLC), is regarded as the most specific and sensitive quantitative method alternative to electron paramagnetic resonance (EPR) for measuring O_2_^∙-^ to date [1]. Typically, excised tissues or cells are incubated *ex vivo* with DHE [2, 3]. Nevertheless, there have been studies describing the topical administration of DHE to murine carotid arteries [4], where DHE was applied *in vivo* intraperitoneally [5–11] or intravascularly [12, 13] to study oxidative stress *ex vivo* in the brain using DHE-derived red fluorescence as a marker of O_2_^∙-^ production; unfortunately, this is not a specific method of O_2_^∙-^ detection [14]. Moreover, subcutaneous injection of DHE has been used to study O_2_^∙-^ in various tissues with fluorimetric detection [15, 16]; however, the authors did not take into account that the major fluorescent product of DHE oxidation is ethidium (E^+^) nor did they elucidate the abundance of the parent compound in analyzed tissues.

The aim of this study was to assess the feasibility of multiorgan *in vivo* oxidative stress detection using intravenously injected DHE, with subsequent quantitative HPLC-based analysis of the tissue accumulation of DHE, 2-OH-E^+^, and E^+^ and their ratios. To evaluate this methodology, we used mice with lipopolysaccharide- (LPS-) induced endotoxemia, as this model is well characterized by an inflammatory reaction with an NADPH-oxidase-dependent oxidant burst and elevation in RNS through the inducible nitric oxide synthase (NOS-2) pathway [17]. The LPS insult is known to increase the level of proinflammatory cytokines [18, 19], which leads to excessive production of nitric oxide (^∙^NO) via NOS-2 and increased production of prostanoids due to cyclooxygenase-2 (COX-2) induction as well as concomitant alternative mechanisms leading to endotoxemic multiorgan failure [20]. Since little is known regarding tissue-specific differences in changes of ROS/RNS balance *in vivo* as most studies focused on a single target organ or analysis of proinflammatory cytokines [19, 20], the tissue- and time-dependent changes in DHE oxidation products were supplemented by *ex vivo* detection of ^∙^NO production using EPR spin trapping in the isolated aorta, lung, and liver tissues. Additional analysis from the blood was used to validate endotoxemia severity for reliable interpretation.

## 2. Materials and Methods

### 2.1. Animal Experimental Protocol

Thirty male 3-month-old C57Bl/6 mice purchased from Lodz University (Poland) were randomly assigned into three experimental groups (control, LPS 6 h, and LPS 12 h), housed 5 per cage, and maintained at 22-24°C under a 12-hour light/day cycle with *ad libitum* access to water and rodent chow. To induce endotoxemia, LPS (from *Salmonella typhosa*, Sigma-Aldrich, St. Louis, MO, United States) was injected intraperitoneally (10 mg/kg) and mice were sacrificed six (*n* = 10) or twelve (*n* = 10) hours later. Control animals (*n* = 10) were treated with intraperitoneal injections of adequate volumes of saline six hours before sacrifice. All mice received an injection of DHE (10 mg/kg, 3 mg/mL, 40% DMSO in PBS, in a roughly 90 *μ*L injection) through the tail vein one hour before anesthetization with ketamine and xylazine (100 mg/kg and 10 mg/kg, respectively, i.p. Pfizer, New York, NY, United States). At the time of sacrifice, the mouse chest was surgically opened, blood was taken from the right ventricle into syringes containing heparin (10 U/mL), and animals were perfused via the left and right ventricles with ice-cold PBS for a total of 10 minutes. Due to the light sensitivity of DHE [21], intravenous injections as well as blood collection and tissue harvest were conducted under low-light conditions. All experimental procedures were compliant with the Guide for the Care and Use of Laboratory Animals published by the U.S. National Institutes of Health (NIH Publication No. 85-23, revised 1996) and were approved by the First Local Ethical Committee on Animal Experiments at the Jagiellonian University in Krakow, Poland (permit no: 19/2016), in accordance with the Guidelines for Animal Care and Treatment of the European Community.

### 2.2. HPLC-Based Detection of DHE and Oxidation Products Formed In Vivo in Tissues

Immediately after perfusion, tissues (lung, liver, kidney, and brain) were dissected, drained on a piece of Kimwipe, snap-frozen in liquid nitrogen in light-safe tubes, and stored at -80°C until analyzed. On the day of HPLC analysis, the samples were thawed on ice and homogenized, and DHE along with the oxidation products were extracted and analyzed, as described previously [1] with minor modifications [22]. Results were normalized to the protein content of each sample, assessed in the initial supernatant, unless stated otherwise.

### 2.3. Nitric Oxide Production Assessed Ex Vivo Based on EPR Spin Trapping

Isolated aorta, liver, and lung tissues were used for the measurement of ^∙^NO production by EPR spin trapping with diethyldithiocarbamic acid sodium salt (DETC) as described previously [23] with minor modifications. Briefly, endothelial nitric oxide synthase- (NOS-3-) dependent production of ^∙^NO was measured by incubating isolated and cleaned aortic tissue (*n* = 5 per group) with 350 *μ*l Fe(DETC)_2_ colloid (final concentration 285 *μ*M) with the addition of calcium ionophore A23187 stimulation (final concentration 1 *μ*M). Isolated lung lobe and liver pieces were incubated similarly but without stimulation, to measure the NOS-2-dependent nitric oxide production. After 90 min of incubation, each tissue was removed from the buffer, drained on a piece of Kimwipe for 5 s, and its wet mass measured. Measurement of the NO-Fe(DETC)_2_ signal in the frozen samples was performed in a finger Dewar (Noxygen Science Transfer & Diagnostics GmbH, Germany) using an EMX Plus Bruker spectrometer (Bruker BioSpin GmbH, Silberstreifen, Rheinstetten, Germany) with the following settings: microwave power, 10 mW; modulation amplitude, 0.8 mT; scan width, 11.5 mT; scan time, 61.44 s; and number of scans, 4. The resulting spectra were collected and analyzed with WinEPR Processing software (Bruker BioSpin GmbH). The NO-Fe(DETC)_2_ signal was a clear triplet centered at *g* = 2.039 and was quantified as the amplitude normalized to the weight of tissue.

### 2.4. Quantification of Select Eicosanoids in Plasma

The quantification of prostaglandin E_2_ and D_2_ (PGE_2_ and PGD_2_), thromboxane B_2_ (TXB_2_), and 12-hydroxyeicosatetraenoic acid (12-HETE) was performed using the liquid chromatograph UFLC Nexera (Shimadzu, Kyoto, Japan) coupled to the triple quadrupole mass spectrometer QTRAP 5500 (SCIEX, Framingham, MA, USA). Samples were purified via a liquid-liquid extraction technique using 0.5 mL of ethyl acetate acidified with 0.13% of AA (*v*/*v*). After 10 minutes of shaking (1500 rpm), 0.45 mL of the organic layer was evaporated to dryness under a nitrogen stream at 37°C. The dry residue was reconstituted in 50 *μ*L EtOH, and samples were injected onto an ACQUITY UPLC BEH C18 analytical column (3.0 × 100 mm, 1.7 *μ*m; Waters, Milford, MA, USA). The mobile phase consisted of 0.1%FA in ACN (a) and 0.1%FA in water (*v*/*v*) (b), which was delivered at the flow rate of 350 *μ*L/min. The detection of PGE_2_, PGD_2_, TXB_2_, and 12-HETE as well as their internal standards was carried out by applying negative ion electrospray ionization mass spectrometry in the multiple reaction monitoring mode (MRM). Data acquisition was performed under the following optimized conditions: spray voltage: -4500 V, source temperature: 500°C, curtain gas: 25 psi, ion source gas 1: 40 psi, and ion source gas 2: 50 psi.

### 2.5. Measurement of Plasma Xanthine Oxidase Activity

The enzymatic activity of xanthine oxidase (XO) in plasma was determined either spectrophotometrically by the rate of uric acid formation monitored at 292 nm in 50 mM potassium phosphate (KPi), pH 7.4 (*ε* = 11 mM^−1^ cm^−1^) or electrochemically via reverse phase HPLC analysis of uric acid production (ESA CoulArray System, Chelmsford, MA) (1 Unit = 1*μ*mole urate/min) as described previously [24].

### 2.6. Biochemical Assays

Blood cell count was determined by the hematology analyzer ABC Vet (Horiba, Germany). Plasma obtained by centrifugation at 1000 ×g for 5 min at 4°C was transferred into Protein LoBind tubes, split into 50 *μ*L aliquots, and stored at −80°C until further use. Alanine aminotransferase (ALT), aspartate aminotransferase (AST), and creatinine were measured using Pentra 400 (Horiba, Kyoto, Japan), according to the manufacturer's instructions. Systemic nitric oxide production was characterized by nitrite (NO_2_^−^) and nitrate (NO_3_^−^) concentrations in plasma using the ENO-20 NOx Analyzer (Eicom Corp., Kyoto, Japan) as described previously [25]. Mouse serum amyloid A (SAA) was measured in heparinized plasma using an enzyme-linked immunosorbent assay kit (Phase SAA Murine Assay Kit, cat. No. TP-802M, Tridelta Development Ltd.), according to the manufacturer's protocol.

### 2.7. Statistical Analysis

Data are expressed as mean and SD or median and interquartile ranges (Q1-Q3, IQR), depending on data distribution and homogeneity of variance, tested with Shapiro-Wilk's and Bartlett's or *F* tests. The significance of differences was tested using one-way ANOVA or the Kruskal-Wallis nonparametric test with appropriate post hoc multiple comparison tests using GraphPad Prism 7 (GraphPad Software Inc., CA, USA). *p* values <0.05 were considered statistically significant.

## 3. Results

### 3.1. Tissue-Specific In Vivo Oxidative Stress in Endotoxemia

**Figure 1:**
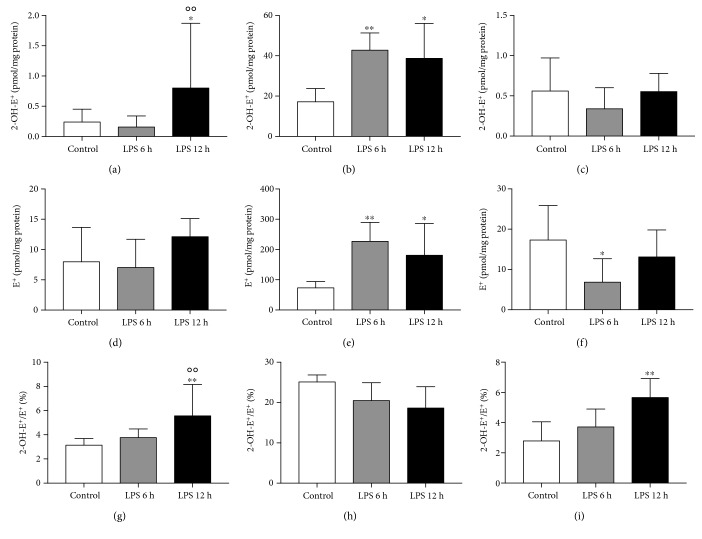
Monomeric DHE oxidation products formed *in vivo* in the liver (a, d, and g), lung (b, e, and h), and kidney (c, f, and i) tissues in a murine model of endotoxemia, 6 and 12 hours after LPS challenge. Using HPLC-Fl detection, the superoxide-specific 2-OH-E+ and nonspecific E+ monomeric products of DHE oxidation were measured according to the details presented in Materials and Methods. Additionally, the ratio of 2-OH-E+/E+ was calculated for each sample. Data are presented as mean ± SD or median ± IQR, and statistical significance was tested using one-way ANOVA or the nonparametric Kruskal-Wallis ANOVA, depending on the distribution and homoscedasticity of data. *p* values <0.05 were considered significant, with ∗ <0.05 and ∗∗ <0.01 versus the control group and ○○ <0.01 versus the LPS 6 h group.

**Figure 2:**
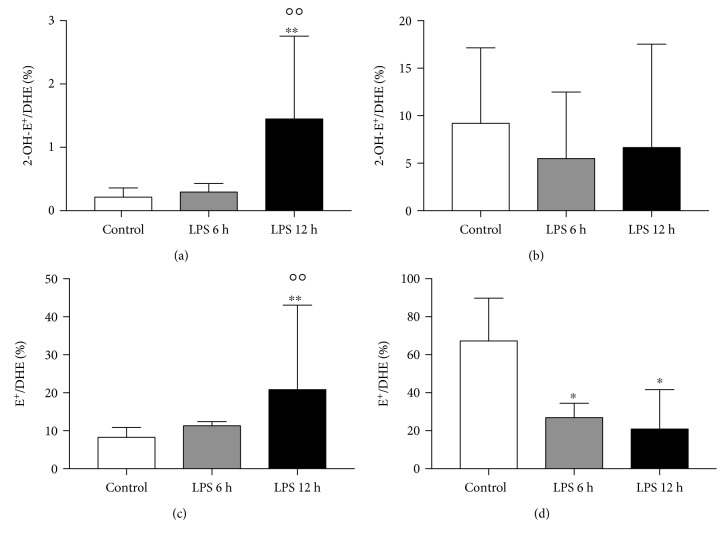
Superoxide-specific and unspecific oxidation of DHE accumulated in the liver and lung tissue in a murine model of endotoxemia, 6 and 12 hours after LPS challenge. Ratios of 2-OH-E^+^/DHE accumulated in the liver and lung tissues suggest that the liver is under increased superoxide-specific oxidant stress (a), as opposed to the lung (b). Moreover, the ratio of E^+^/DHE also suggests increased superoxide-independent oxidative stress in the liver (c), which is not the case in the lung (d). Data are presented as median ± IQR, and statistical significance was tested using nonparametric Kruskal-Wallis ANOVA. *p* values <0.05 were considered significant, with ^∗^<0.05 and ^∗∗^<0.01 versus the control group, and <0.01 versus the LPS 6 h group.

Using HPLC with fluorescent detection, the accumulation of DHE injected *via* the tail vein and its superoxide-specific and nonspecific oxidation products (2-OH-E^+^ and E^+^, respectively) was quantified in the liver, lung, kidney, and brain tissues (Table 1) in LPS-injected and control mice. The parent DHE compound was detected in the liver and lung tissue of control mice in similar amounts but was below the limit of detection (LOD) in both kidney and brain. After the LPS challenge, the DHE delivery to the liver was unaffected; however, there was increased accumulation in the lungs, which was statistically significant at 6 hours post-injection. To compare tissues independently from their pharmacokinetic profile, the ratio of 2-OH-E^+^/E^+^ was calculated for each sample. In the liver, elevation of the 2-OH-E^+^ level was observed (Figure 1(a)), indicating increased O_2_^∙-^-specific oxidation 12 h after LPS challenge, whereas E^+^ content remained unchanged (Figure 1(d)). The 2-OH-E^+^/E^+^ ratio confirmed superoxide-dependent oxidative stress in the liver 12 h after LPS challenge (Figure 1(g)). In the lung, there were increases in both 2-OH-E^+^ and E^+^ concentrations 6 h and 12 h post-LPS injection (Figure 1(b) and 1(e), respectively). In turn, there was no statistical difference in the 2-OH-E^+^/E^+^ ratio (Figure 1(h)). In the kidney, 2-OH-E^+^ and E^+^ concentrations were unchanged (Figure 1(c) and 1(f), respectively); however, a gradual increase in the 2-OH-E^+^/E^+^ ratio was appreciated (Figure 1(i)), which attained statistical significance 12 h post-LPS injection. In the brain, there was no 2-OH-E^+^ found in any of the samples from the control group and only a few samples were above the limit of detection in LPS-injected mice (3 among 10 for the 6 h group and 1 among 10 for the 12 h group); however, E^+^ was detected in all samples. The 2-OH-E^+^/E^+^ ratio was unchanged between control and LPS-treated mice. Where applicable (liver and lung), the ratios of 2-OH-E^+^/DHE and E^+^/DHE were also calculated to quantify the amount of oxidation products in relation to DHE deposition in particular samples (Figure 2).

### 3.2. Tissue-Specific Ex Vivo Nitric Oxide Production and Plasma Nitrate/Nitrite Concentration in Endotoxemia

**Figure 3:**
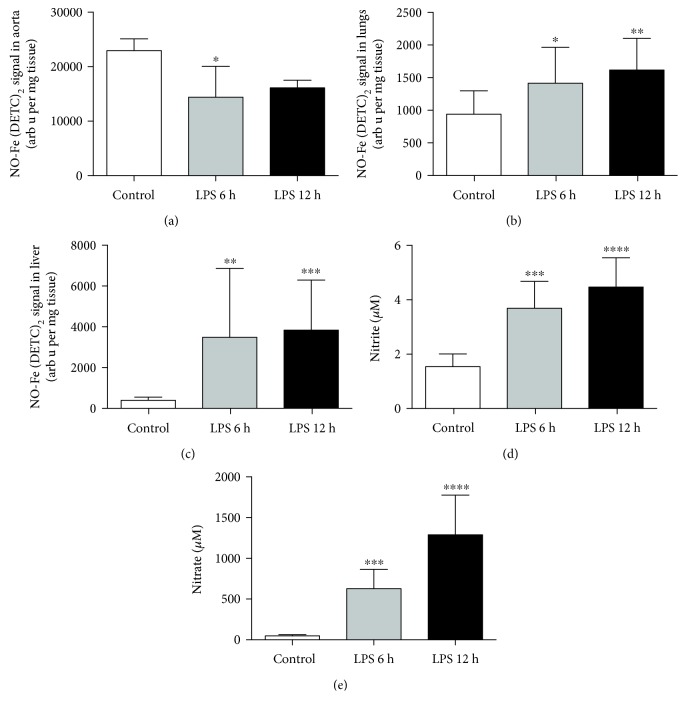
Nitric oxide production *ex vivo* in tissues and nitrite/nitrate burst in plasma in a murine model of endotoxemia, 6 and 12 hours after LPS challenge. EPR spin trapping as described in Materials and Methods was used to detect nitric oxide *ex vivo* in the aorta, lung, and liver tissues (a, b, and c, respectively), whereas nitrite (d) and nitrate (e) were measured in the plasma using the ENO-20 apparatus. Gradually increased NO production was seen in the lungs at 6 and 12 hours post-LPS injection (b), while the liver showed a much higher NO overproduction (c, 6-7-fold change). Data are presented as mean ± SD or median ± IQR (in c), and statistical significance was tested using one-way ANOVA post hoc LSD's test or Kruskal-Wallis's, post hoc Dunn's (in c), depending on the distribution and homoscedasticity of data. *p* values < 0.05 were considered significant, with ∗ <0.05, ∗∗ <0.01, ∗∗∗ <0.005, and ∗∗∗∗ <0.001 versus the control group.

In the aorta, decreased ionophore-stimulated ^∙^NO production was detected (Figure 3(a)) post-LPS, which was compatible with impaired NOS-3-dependent nitric oxide production and endothelial dysfunction. A moderate and progressive increase in ^∙^NO production was detected in the lung (Figure 3(b)), while a strong nitric oxide burst was noted in the liver (Figure 3(c)), compatible with the differential extent of NOS-2 induction in the liver as compared with the lung. Likewise, nitrite and nitrate (Figures 3(d) and 3(e)) levels were both progressively elevated compared to those of the control mice; however, a statistically significant difference between 6 and 12 hours after LPS administration was found only for nitrate.

### 3.3. Biochemical Markers in Plasma and Blood Count Changes in Endotoxemia

**Figure 4:**
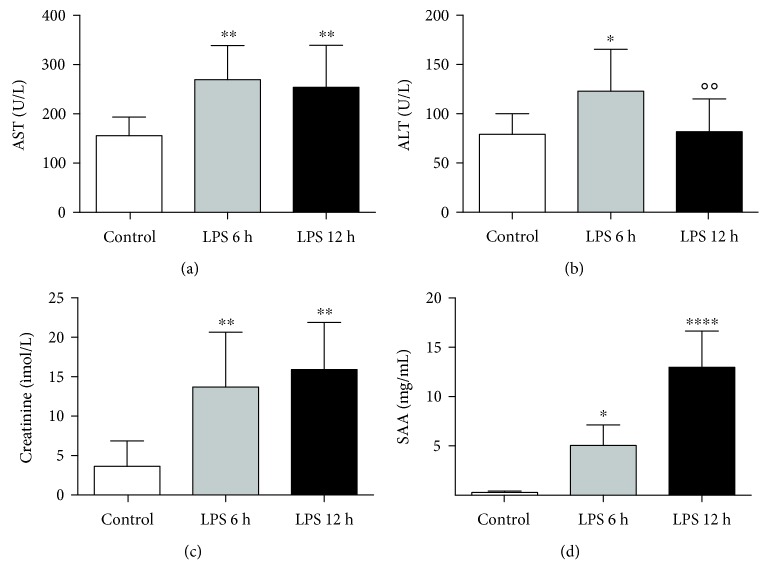
Markers of the organ injury and systemic inflammation in a murine model of endotoxemia, 6 and 12 hours after LPS challenge. Liver damage—ALT (a) and AST (b), kidney damage—creatinine (c), and systemic inflammation—SAA (d). Data are presented as mean ± SD or median ± IQR, and statistical significance was tested using one-way ANOVA post hoc LSD's test or Kruskal-Wallis, post hoc Dunn's, depending on the distribution and homoscedasticity of data. *p* values <0.05 were considered significant, with ∗ <0.05, ∗∗ <0.01, and ∗∗∗∗ <0.001 versus the control group and ○ <0.05 and ○○ <0.01 versus the LPS 6 h group.

**Figure 5:**
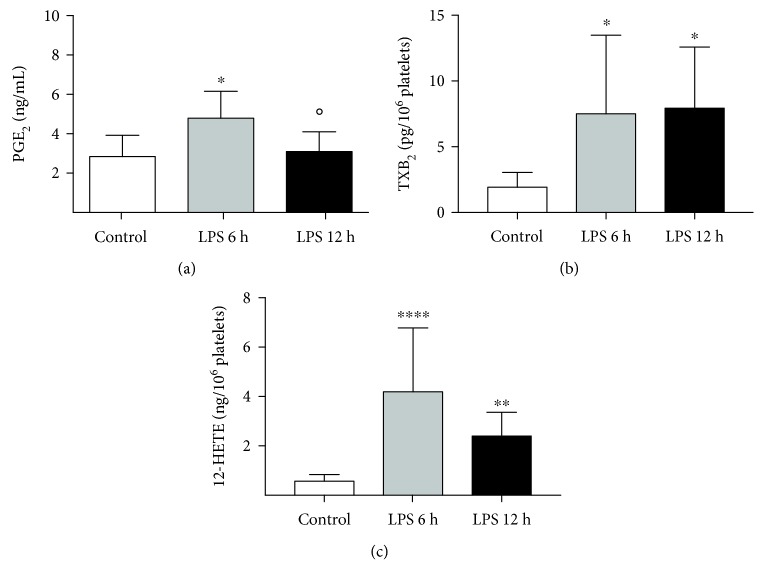
Eicosanoid concentration in plasma in a murine model of endotoxemia, 6 and 12 hours after LPS challenge. PGE_2_ (a), TXB_2_ (b), and 12-HETE (c). Data are presented as mean ± SD or median ± IQR, and statistical significance was tested using one-way ANOVA post hoc LSD's test or Kruskal-Wallis, post hoc Dunn's (for b, c), depending on the distribution and homoscedasticity of data. *p* values <0.05 were considered significant, with ^∗^ <0.05, ^∗∗^ <0.01, and ^∗∗∗∗^ <0.001 versus the control group, and ○ <0.05 and ○○ <0.01 versus the LPS 6 h group.

The development of endotoxemic inflammation after injection of LPS was confirmed by dramatically increased SAA, 6 h after LPS injection that further increased 12 h after LPS challenge and was associated with a significant increase in plasma biomarkers of liver (ALT, AST) and kidney (creatinine) injuries (Figure 4) as well as blood count changes (Table 2). The LPS-induced changes in the eicosanoid profile (Figure 5) encompassed elevation of plasma concentration of PGE_2_, without changes in PGD_2_, but increased TXB_2_ and 12-HETE concentration in plasma. Furthermore, an approximately 5-fold significant (*p* < 0.0007, Kruskal-Wallis ANOVA, post hoc Dunn's test) and sustained increase in plasma xanthine oxidoreductase activity was found in LPS-challenged mice (6 h: 2.0, 1.3-2.7 and 12 h: 1.9, 1.4-2.8, in mU/mL) when compared with the control group (0.4 and 0.2-0.6 mU/mL).

## 4. Discussion

In the present work, taking advantage of the HPLC-based methodology for the assessment of oxidative stress and O_2_^∙-^ production *in vivo,* based on the quantification of DHE uptake and monomeric oxidation product formation (2-hydroxyethidium (2-OH-E^+^) and ethidium (E^+^)) combined with *ex vivo* EPR spin trapping of nitric oxide production, we demonstrated the differential progression and heterogeneity of oxidative and nitrosative stress in endotoxemia in various organs. LPS-induced inflammation was confirmed by several biomarkers [17] including blood count, SAA, and eicosanoids (Table 2, Figures 4 and 5). It is important to note that DHE was administered intravascularly to overcome impaired splanchnic microcirculation in endotoxemia, which could have disturbed the uptake of DHE via the hepatic portal system following intraperitoneal injection of DHE used previously [5–11, 26].

Results demonstrated variable distribution of DHE, with the lung exhibiting the greatest uptake followed by the liver, kidney, and brain. In the control group, the lung exhibited a 100-fold greater accumulation of 2-OH-E^+^ and 5-10-fold greater accumulation of E^+^ compared to the other three tissues (Table 1), suggesting that the lung represents a major target for DHE following i.v. injection. After LPS administration, the lung has shown a 6-7-fold increase in DHE accumulation, most likely due to increased pulmonary vascular permeability, as reported in acute lung injury [27]. Furthermore, there was roughly a 2-fold increase in both 2-OH-E^+^ and E^+^ detected in the lungs after LPS challenge. This alone would suggest robust oxidative stress in the lung in endotoxemia, and due to an unaltered 2-OH-E^+^/E^+^ ratio, one could speculate that both superoxide-dependent and superoxide-independent mechanisms are responsible. However, with DHE also measured and the 2-OH-E^+^/DHE ratio unchanged along with reduced E^+^/DHE ratio (Figure 2(b) and 2(d), respectively), this would rather suggest that during endotoxemia in the lung, the single electron oxidation of DHE might be shifted from disproportionation, which leads to E^+^ formation, towards dimerization, resulting in the formation of dimeric products [14], though verification of this hypothesis remains beyond the scope of this article. A different distribution pattern was observed in the liver where DHE, 2-OH-E^+^, and E^+^ accumulation did not change upon LPS challenge, despite a systemic increase in vascular permeability in endotoxemia [28] and dysfunctional hepatic circulation post-LPS injection. Nevertheless, the 2-OH-E^+^/E^+^ ratio increased significantly 12 hours post-LPS challenge (Figure 1(g)), similar to both 2-OH-E^+^/DHE and E^+^/DHE ratios (Figure 2(a) and 2(c), respectively), suggesting that the liver is under O_2_^∙-^-dependent and O_2_^∙-^-independent oxidative stress. With no DHE detected in the brain and kidney, the origin of the detected 2-OH-E^+^ and E^+^ remains unclear. These could have accumulated from the circulation, as the blood-brain barrier integrity after LPS is compromised [28]. Importantly, only E^+^ was found in the brain tissue, suggesting that the previously reported oxidative stress using DHE fluorescence in the brain [5–13] may not have been formed *in situ*. On the other hand, DHE could have reached these tissues but was not detectable due to complete oxidation.

Inhomogeneous DHE organ distribution and various contents of oxygenation products in organs demonstrate the value of our approach. This was based on the normalization of 2-OH-E^+^ abundance to the amount of DHE accumulated in the tissue or to the amount of E^+^ formed, assuming E^+^ also reflects the intracellular uptake of DHE [29]. The latter approach has shown superoxide-driven oxidative stress in the liver and kidney 12 hours after injection of LPS (Figures 1(d), 1(g) and 1(f), 1(i), respectively) but not in the lung (Figure 1(e) and 1(h)), questioning the reliability of oxidative stress assessment based solely on 2-OH-E^+^ measurements. In fact, after LPS the lung exhibited increased deposition of DHE monomeric oxidation products as well as the parent compound. Calculation of the ratio of 2-OH-E^+^/DHE in the lung samples, unaffected by LPS administration, suggests that the increase in 2-OH-E^+^ contents is related to higher DHE accumulation after LPS (Figure 2(b)). Interestingly, the ratio of E^+^/DHE (Figure 2(d)) significantly decreased in the lungs of LPS-treated mice, suggesting that in these conditions the dimeric products of DHE oxidation might be favorable perhaps due to cytochrome c activity, which is known to promote DHE oxidation to the dimeric products over ethidine [14]. Alternatively, the lower ratio of E^+^/DHE might be due to increased hydrogen peroxide (H_2_O_2_) production in the lungs, which could oxidize cytochrome c and thus generate cytochrome c redox cycling [30], in consequence producing more superoxide and oxidizing DHE to 2-OH-E^+^ in favor of E^+^. Similar analysis for the liver showed increased ratios of both 2-OH-E^+^/DHE and E^+^/DHE twelve hours after LPS injection, suggesting that both superoxide and other ROS contribute to oxidant stress in the liver (Figure 2(a) and 2(c)), respectively. In fact, xanthine oxidoreductase (XOR) activity was increased in the plasma of LPS-treated mice (6 h: 2.0, 1.3-2.7 and 12 h: 1.9, 1.4-2.8, in mU/mL vs. 0.4, 0.2-0.6 mU/mL in control), suggesting that XOR-catalyzed H_2_O_2_ production, the major ROS product of XOR under physiological and hypoxic O_2_ tensions [31], might be relevant. However, dimeric DHE oxidation products, not measured here due to technical limitations, have also been associated with H_2_O_2_/heme protein reactions [32].

Higher levels of oxidation products detected after LPS might be related to amplified DHE uptake caused by increased permeability, as shown in LPS-induced acute lung injury by [27] along with increased NO production in this tissue (Figure 3(b)), rather than increased oxidative stress itself. Another plausible explanation for increased E^+^ concentration in the lung and liver tissue is peroxynitrite (O=NOO^−^) formation that occurs when both ^∙^NO and O_2_^∙-^ production are increased. In fact, peroxynitrite formation and increased protein nitration have previously been reported in the lung and liver in LPS-challenged mice [33, 34]. Our methodology for ^∙^NO detection in the lung and liver tissues did not use exogenous stimuli; therefore, EPR spin trapping results reflect the basal ^∙^NO production with a fairly low level of ^∙^NO production in the control group. However, after LPS administration the level of ^∙^NO produced in the lung tissue significantly increased at 6 h and even to a greater extent at 12 h post-injection. As shown in a rat model of lung inflammation by [35], LPS induces NOS-2 but not NOS-3 or NOS-1 in the lung tissue, which upholds our conclusion. Nevertheless, the nitric oxide increase observed in the lung is rather moderate compared to the effects LPS had on nitric oxide production in the liver (Figure 3(c)), which can be accounted to NOS-2 activation in hepatocytes as early as 3 h after LPS treatment of an isolated rat liver, as shown in a previous work [36]. Furthermore, we saw increased NOx-species in plasma (Figures 3(d) and 3(e)), altogether suggesting systemic nitric oxide overproduction. In acute inflammation and sepsis, this has been described previously and was shown to provide protection in endotoxemia-induced hepatic injury [37, 38] and initial protection against acute oedema during the first two hours [39]. On the other hand, oxidative/nitrosative stress linked to NOS-2 expression was shown to contribute to lung injury [40], while all nitric oxide synthase isoforms contribute to acute peritonitis [41], showing evidence of organ- and time-dependent ^∙^NO-dependent protection. Based on our experiments, we hypothesise that the high overproduction of ^∙^NO in the liver after LPS injection provided protection from oxidative stress 6 hours after endotoxemia was initiated; however, at 12 hours post-LPS injection it leads to oxidative stress, which is not only superoxide dependent. Interestingly, moderately increased ^∙^NO production in the lung in endotoxemia correlates with increased accumulation of DHE and its oxidation products, but not oxidative stress, expressed as ratios of either 2-OH-E^+^/DHE, E^+^/DHE, or 2-OH-E^+^/E^+^, as proposed herein. Accordingly, our methodological approach with the normalization of the 2-OH-E^+^ to the E^+^ content in tissue demonstrated a distinct ROS environment in the lung and liver after i.p. LPS.

Interestingly and in opposition to the nitrosative stress systemically shown in the lung and liver, we demonstrated decreased ^∙^NO production in the isolated aorta as early as 6 hours after LPS challenge using EPR spin trapping (Figure 3(a)). A decreased NO-Fe(DETC)_2_ signal is indicative of endothelial dysfunction, as seen previously in other rodent models [23, 42], and it is associated with lower NOS-3 activity and altered NOS-3 phosphorylation, as reported previously [43]. The development of peripheral endothelial dysfunction, confirmed by decreased ^∙^NO release from the aorta, was coincident with increased platelet activity evidenced by elevated levels of platelet-derived TXB_2_ (a metabolite of TXA_2_) and 12-HETE during LPS-induced inflammation (Figure 5).

Although HPLC-based detection of DHE oxidation products is one of the more specific methods of detection of the intracellular steady-state superoxide activity, the amount of 2-OH-E^+^ measured can only serve as a semiquantitative measure of superoxide concentration and comparison of absolute values between tissues and conclusions must be made with caution, due to competing reactions with superoxide dismutases, heme proteins, or other exogenous compounds affecting DHE uptake and conversion *in situ* to 2-OH-E^+^ [14]. On the other hand, DHE analysis *in vivo* should not prohibit further mechanistically-oriented analyses using for example Western blots; however, measuring any other redox-dependent outcomes might obviously provide altered results, as DHE neutralizes superoxide. The methodology described in this paper could be used in any experimental model where one would want to study the *in vivo* abundance of superoxide, especially in multiorgan diseases or in the assessment of oxidant stress-targeted therapeutic approaches. Moreover, this methodology could, in principle, be targeted towards the detection of mitochondrial superoxide anions *in vivo* using MitoSOX, instead of DHE.

Recently, a new NS-O two-photon fluorescent probe specific for the superoxide radical anion has been developed and tested in living cells, tissues, and zebrafish [44], which might develop in time as a better alternative to DHE oxidation coupled with HPLC-based detection. Alternatively, immunospin trapping [45] has emerged as an interesting method to study the extent and localization of oxidative modifications *in vivo* and was used in acute endotoxemia [46, 47] as well as in chronic inflammation [48] or in heart failure development [49].

## 5. Conclusions

Our work demonstrates that intravenous injection of DHE with consecutive HPLC analysis of its oxidation products and calculation of 2-OH-E^+^/E^+^ ratios provide a means to quantify and compare oxidative stress in multiple tissues *in vivo*. This method overcomes the limitations of previous studies assessing superoxide production *in vivo* without taking into account differences in the organ bioavailability of DHE and not quantifying the concentrations of specific and nonspecific DHE oxidation products in the organ, as we did here. Results indicate a substantive increase in superoxide-dependent oxidative stress in endotoxemia in the liver and kidney with a moderate impact in the lung during endotoxemic injury induced by i.p. LPS challenge.

## Figures and Tables

**Table 1:** DHE, 2-OH-E^+^, and E^+^ detected in tissues after injection of DHE (i.v.) in a murine model of endotoxemia, 6 and 12 hours after LPS challenge.

	Tissue	Control	LPS 6 h	LPS 12 h
DHE (pg/mg protein)	Liver	104 ± 70**(n** = 6**)**	81 ± 72**(n** = 9**)**	60 ± 15**(n** = 6**)**
	Lung	139 ± 51 (*n* = 4)	920 ± 501**(n** = 7**)**^∗^	782 ± 566**(n** = 4**)**
	Kidney	**<LOD**	<LOD	<LOD
	Brain	**<LOD**	<LOD	<LOD

2-OH-E^+^ (pg/mg protein)	Liver	0.26 ± 0.18**(n** = 6**)**	0.19 ± 0.15 (*n* = 8)	1.18 ± 0.80**(n** = 9**)**^∗^
	Lung	21.1 ± 2.6**(n** = 4**)**	42.8 ± 8.6**(n** = 7**)**^∗∗^	38.7 ± 17.4**(n** = 4**)**^∗^
	Kidney	0.56 ± 0.41**(n** = 8**)**	0.34 ± 0.26 (*n* = 9)	0.55 ± 0.23 (*n* = 9)
	Brain	**< LOD**	0.12 ± 0.04 (*n* = 3)	0.31 (*n* = 1)

E^+^ (pg/mg protein)	Liver	7.9 ± 5.7**(n** = 6**)**	7.0 ± 4.7 (*n* = 8)	12.1 ± 3.9 (*n* = 7)
	Lung	85 ± 14**(n** = 4**)**	227 ± 63**(n** = 7**)**^∗∗^	221 ± 62**(n** = 4**)**^∗^
	Kidney	17.3 ± 8.5**(n** = 8**)**	6.8 ± 5.8**(n** = 9**)**^∗^	13.1 ± 6.7 (*n* = 10)
	Brain	16.1 ± 8.8**(n** = 7**)**	7.6 ± 5.3**(n** = 7**)**^∗∗^	12.9 ± 7.6**(n** = 8**)**^∗^

Data are presented as mean ± SD. Results of Student's*t*-test vs. control (in bold):^∗^*p* < 0.05, ^∗∗^*p* < 0.01, ^∗∗∗^*p* < 0.005, and ^∗∗∗∗^*p* < 0.001.

**Table 2:** Hematologic alterations in endotoxemia, 6 and 12 hours after LPS challenge.

	Control	LPS 6 h	LPS 12 h
WBC (10^3^/*μ*L)	8.51 ± 1.00	4.52±0.61^∗∗∗^	7.69 ± 1.04^#^
RBC (10^3^/*μ*L)	10.26 ± 0.25	9.55 ± 0.15^∗^	8.73±0.31^∗∗∗^^,#^
PLT (10^3^/*μ*L)	1145 ± 55	663±51^∗∗∗∗^	642±51^∗∗∗∗^
GRA (%)	36.6 ± 2.8	50.3±2.3^∗∗∗∗^	65.1±2.1^∗∗∗∗^^,####^
MON (%)	7.14 ± 0.28	10.29 ± 1.07^∗^	8.66 ± 0.53^∗^
LYM (%)	56.3 ± 2.8	39.6±1.6^∗∗∗∗^	25.9±1.8^∗∗∗∗^^,####^
GRA (10^3^/*μ*L)	3.27 ± 0.47	2.30 ± 0.26	5.03 ± 0.75
Hct (10^3^/*μ*L)	56.1 ± 1.4	52.9 ± 1.0	47.6±1.8^∗∗∗^^,#^
HGB (10^3^/*μ*L)	15.43 ± 0.28	14.68 ± 0.29	13.64±0.46^∗∗∗^
LYM (10^3^/*μ*L)	4.69 ± 0.54	1.97±0.31^∗∗∗∗^	1.79±0.15^∗∗∗∗^
MCH (10^3^/*μ*L)	15.08 ± 0.25	15.37 ± 0.14	15.64 ± 0.21
MCHC (10^3^/*μ*L)	27.53 ± 0.28	27.76 ± 0.19	28.71±0.24^∗∗^^,##^
MCV (10^3^/*μ*L)	54.78 ± 0.57	55.30 ± 0.45	54.50 ± 0.67
MON (10^3^/*μ*L)	0.56 ± 0.08	0.61 ± 0.21	0.63 ± 0.12
MPV (10^3^/*μ*L)	5.37 ± 0.12	5.11 ± 0.05	5.50 ± 0.12^##^
RDW (10^3^/*μ*L)	12.2 ± 0.2	12.1 ± 0.2	12.3 ± 0.3

WBC—white blood cells, RBC—red blood cells, PLT—platelets, GRA—granulocytes, MON—monocytes, and LYM—lymphocytes. Results of Student's*t*-test vs. control:^∗^*p* < 0.05, ^∗∗^*p* < 0.01, ^∗∗∗^*p* < 0.005, and ^∗∗∗∗^*p* < 0.001; LPS 6 h vs. 12 h:^#^*p* < 0.05, ^##^*p* < 0.01, and ^####^*p* < 0.001.

## Data Availability

The datasets generated and/or analyzed during the current study are available from the corresponding author on reasonable request.
